# The Use of Laser Photobiomodulation in the Treatment of Oro-Facial Neurosensory Alterations—A Case Series from a University Clinic

**DOI:** 10.3390/healthcare14101283

**Published:** 2026-05-08

**Authors:** Amanda Silva Santos, Maria Cristina Teixeira Cangussu, Antônio Luiz Barbosa Pinheiro, Jean Nunes Dos Santos

**Affiliations:** 1 RENORBIO Postgraduate Program, Institute of Health Sciences (ICS/UFBA), Faculty of Dentistry, Federal University of Bahia (FOUFBA), Santa Isabela Street, 100, Garibaldi, Salvador 40221-225, Bahia, Brazil; 2 Faculty of Dentistry, Federal University of Bahia (FOUFBA), Salvador 40110-040, Bahia, Brazil; cangussu@ufba.br (M.C.T.C.); albp@ufba.br (A.L.B.P.); jeanpatol@gmail.com (J.N.D.S.)

**Keywords:** paresthesia, low-level light therapy, biotechnology

## Abstract

**Objective:** To describe the experience of use of laser photobiomodulation in the management of orofacial neurosensory alterations and its clinical outcomes in a series of cases at a public university clinic in Northeast Brazil. **Methods:** A retrospective case series study was conducted using secondary data from 125 patients treated at the Biophotonics Center of the Federal University of Bahia between 2003 and 2019, all with a confirmed diagnosis of orofacial neurosensory alterations and who were referred to the service by other primary healthcare units in the municipality. Data collection included sociodemographic characteristics, clinical and dental history, and the main patient complaint. The therapeutic protocol consisted of infrared diode laser application (λ 700–808 nm) in continuous mode, performed every 48 h, totaling 12 individualized sessions per treatment cycle. Sensory evolution was monitored using the inverted Visual Analog Scale (VAS). Patients quantified their perceived sensitivity and discomfort at each session, assigning values from 0 to 10, and monitored symptom progression and treatment response over time. Data were analyzed using descriptive and analytical approaches. The study was approved by the Research Ethics Committee of UFBA (protocol 60327516500005024). **Results:** Tooth extraction was the most frequently reported etiological factor (60%), with an average of three treatment cycles per patient. At the end of follow-up, 67% of individuals reported changes in symptoms, including partial or complete resolution. Cases associated with orthognathic surgery required more treatment cycles; however, no statistically significant association was observed between the number of cycles and the reported outcomes. **Conclusions:** Laser photobiomodulation has been employed as a therapeutic approach in the management of orofacial neurosensory alterations in this clinical setting. The findings describe patterns of use and the distribution of patient-reported outcomes. Given the observational design, the results do not allow for causal interpretation or inference regarding treatment effectiveness.

## 1. Introduction

Orofacial sensory disturbances, such as paresthesia or dysesthesia, may compromise essential functions, including phonation, mastication, and social interaction [1,2]. Paresthesia is a neurosensory disorder resulting from nerve injury that may occur after dental interventions and is characterized by prolonged loss of sensitivity in a specific region of the face, often accompanied by other symptoms. This condition may resolve spontaneously after a few days or months; however, depending on the extent and type of nerve injury, it may persist. In more severe cases, patients may also report altered tactile perception, burning sensations, or discomfort [2,3]. On the other hand, dysesthesia is an abnormal and often unpleasant sensation that may occur spontaneously or be triggered by normally non-painful stimuli. It is commonly described as burning, tingling, or electric shock-like discomfort, frequently associated with peripheral or central nerve dysfunction. Both conditions may significantly interfere with daily activities and patient well-being and negatively impact quality of life [1,3]. Furthermore, psychological distress and functional limitations are frequently associated with chronic presentations, reinforcing the importance of early diagnosis and appropriate therapeutic management.

These neurosensory disorders have been reported following dental interventions such as third molar extractions [3], implant placement [4,5], local anesthesia [6,7], and orthognathic surgeries [8], as well as facial trauma [9]. The management of these conditions remains challenging due to the persistence and variability of symptoms. Conventional approaches—including pharmacological, surgical, and physiotherapeutic interventions—have been described, although reports indicate heterogeneous responses. Pharmacological management often involves the use of analgesics, anti-inflammatory drugs, anticonvulsants, and antidepressants aimed at modulating neuropathic pain pathways. In selected cases, surgical interventions such as nerve decompression or repair may be indicated, particularly when structural damage is identified. Additionally, physiotherapeutic strategies, including sensory re-education and low-level stimulation techniques, have been employed to promote neural recovery and functional improvement, with variable clinical outcomes [5,7,9]. In this context, alternative approaches such as laser photobiomodulation have been increasingly reported in clinical settings.

Light amplification by stimulated emission of radiation (LASER), developed in the 1960s by Theodore Maiman [5], was promptly identified as a technology with broad potential in medicine, and its various forms are now widely applied across different clinical contexts. Low-level laser (LLL) emits monochromatic, coherent light at a single wavelength and exerts anti-inflammatory effects through a photochemical mechanism known as photobiomodulation therapy (PBMT) [6]. This therapeutic approach can enhance tissue repair by stimulating neovascularization while reducing inflammation and pain through its modulatory effects on cellular activity.

LLL delivers low energy output and has attracted considerable interest among dental professionals for these benefits, particularly its capacity to alleviate muscle spasms [7]. The application of LLL within the wavelength range of 780 to 980 nm is regarded as safe and effective, as it promotes additional photochemical reactions at the mitochondrial level. These reactions contribute to modifications in cellular metabolism and protein synthesis while also ensuring adequate tissue penetration and providing relief in chronic pain conditions [8,9,10,11,12,13,14,15,16].

Laser photobiomodulation has been described as a possible therapeutic approach for the management of orofacial sensory disturbances. Previous studies have reported its clinical application [17,18,19,20,21,22].

Photobiomodulation therapy (PBMT) demonstrates robust results in accelerating peripheral nerve regeneration and restoring neural function. Studies indicate that PBMT promotes an increase in the number of myelinated fibers, better organization of the myelin sheath, and the stimulation of growth factor release. Furthermore, improvements in electrophysiological function and reductions in inflammatory processes and pain associated with the injury are observed [12,16,18].

In clinical applications for maxillofacial trauma, wavelengths between 660 nm and 808 nm led to significant sensory recovery. Research involving patients showed that, after 10 treatment sessions, there was a drastic reduction in paresthesia levels, as measured by the Visual Analog Scale (VAS), and an increase in positive tactile sensitivity responses in brush stroke direction (BSD) tests [12,16].

PBMT also demonstrated a direct impact on quality of life, elevating patients’ perceptions of health and eliminating daily functional difficulties, such as accidental cheek biting and speech or mastication issues. It is concluded that the therapy is a safe and effective resource, capable of providing faster and higher-quality rehabilitation for individuals with orofacial neurological disorders [12,16,18,20].

There is a growing consensus that photobiomodulation is a safe, non-invasive modality with potential adjunctive benefits, particularly in reducing pain and enhancing neural repair. Many studies support its role in improving clinical symptoms and accelerating functional recovery when applied in appropriate therapeutic windows. However, important divergences remain regarding the standardization of treatment parameters, including wavelength, energy density, application frequency, and total dose. Additionally, variability in study designs, sample sizes, and outcome measures contributes to inconsistent evidence, with some authors questioning the magnitude and durability of its clinical effects. Therefore, this study aims to describe the use of defined phototherapeutic protocols in individuals with those conditions in a series of cases in a university clinic and explore patient-reported outcomes.

## 2. Methods

A retrospective study was conducted in a series of cases, using the clinical records of patients treated at the Laser Clinic of the School of Dentistry of the Federal University of Bahia, in Salvador, BA, Brazil, who underwent laser photobiomodulation for the treatment of orofacial sensory disorders (dysesthesia and paresthesia). The study period spanned 2003 to 2019. As this is a referral service within the municipal health network, patients were referred by other primary healthcare units in the municipality, and after completing their course of care, they were returned to these units for follow-up; therefore, the data refer only to individual point-in-time outcomes.

The study population consisted of individuals aged 18 to 90 years, referred to dental clinics or spontaneously through the Public Health System (SUS). All patients with a prior diagnosis and who had a complete clinical record were included. Individuals who did not consent to participate or who abandoned treatment before completing a therapeutic cycle (12 sessions) were excluded. Records with incomplete or unlocatable data were also discarded. The database was structured in Microsoft Excel^®^ (software version 2010, Microsoft Corporation, Redmond, WA, SUA) and after applying the specific filter for R20.2 (ICD-10), the study population totaled 387 cycles corresponding to 125 patients.

The research was approved by the Research Ethics Committee of FO-UFBA (CONEP: 60327516500005024). In compliance with CNS Resolutions 466/12 and 510/16, all participants signed the Informed Consent Form (ICF). Biosafety protocols included the use of Personal Protective Equipment (PPE), rigorous asepsis, compliance with safety standards for laser technology, and the use of specific protective eyewear for the wavelength used, along with adequate signage in the clinical environment.

Experienced professionals conducted the assessments in a standardized outpatient setting. Data collection included anamnesis, extra- and intraoral clinical examinations to identify orofacial alterations, and evaluation of nerve sensitivity using an adaptation of the Visual Analog Scale (VAS). Its use to measure neurosensory alterations is a feasible and pragmatic approach, particularly in clinical settings where rapid, patient-centered assessments are needed. Traditionally used to quantify pain intensity, the VAS can be expanded to capture subjective dimensions of neurosensory disturbances, such as numbness, tingling, burning, or altered sensitivity. In this context, patients are asked to rate the intensity or discomfort associated with these sensations on a continuous scale, thereby quantifying individual perceptions. There is some consensus that the VAS is simple, low-cost, and easily reproducible, supporting its applicability for monitoring treatment response over time. The independent variables collected were: Sociodemographic: Gender and age (categorized by median: 19 to 83 years). Clinical: Etiology, medical/dental history, comorbidities, and use of adjuvant drugs (B complex vitamins and anti-inflammatories). The period was dichotomized by median into 2003–2014 and 2015–2019 for trend analysis.

Photobiomodulation protocols were individualized based on clinical severity under the supervision of the clinic’s coordinator. Data on Dose per Session (DS), Cyclic Dose (CD), number of sessions, laterality, and wavelength (λ) were recorded. The outcome analysis was based on VAS evolution, categorizing results as “asymptomatic” (complete sensory reestablishment), “improvement,” or “symptomatic” (persistence of the condition). From 2018 onwards, retrospective observations were complemented by direct contact between the researcher and the patients, thereby validating the tabulated data.

Clinical Characterization and Etiological Variables: The injured nerve component was determined based on the main complaint and the topography of the symptomatology. Cases involving the inferior alveolar nerve (IAN) included alterations in the lower lip, chin, and body of the mandible. At the same time, sensory alterations in the tongue identified lesions in the lingual nerve (NL). The etiology was established through dental history, categorizing the episodes as resulting from tooth extractions, implant placements, orthognathic surgeries, or facial trauma. Additionally, medical history information allowed stratification of individuals by comorbidities and the use of adjuvant drugs, specifically vitamin B complexes and anti-inflammatory medications, to reduce edema and facilitate nerve decompression.

Dosimetric Parameters and Therapeutic Protocols: The therapeutic regimen consisted of 12-session cycles. For analysis purposes, treatment was classified as a “complete cycle” for those who completed all 12 applications and “incomplete/abandonment” for cases of premature interruption. Dosimetry was analyzed from two perspectives: Dose per Session (DS), defined as the sum of the doses applied at each point in each treatment, and Cyclic Dose (CD), calculated by multiplying DS by the total number of sessions (CD = DS × Total Sessions). Due to the retrospective nature of the secondary data, the number of application points and the specific point dose were not individualized in the database.

Sensory evolution was monitored subjectively using the inverted Visual Analog Scale (VAS). Patients quantified their sensitivity and discomfort in each session, assigning values from 0 to 10. The outcome was determined by the predominant numerical frequency throughout the 12-session cycle, categorizing the results as: Asymptomatic (Scores 8 to 10): Restoration of sensitivity and absence of discomfort; Improvement (Scores 6 to 7): Satisfactory clinical evolution, but with the presence of residual symptoms; Symptomatic (Scores 0 to 5): Persistence of alterations and absence of sensitivity. Individuals who interrupted treatment for more than 30 days were classified as “dropouts”. For inferential analyses, the categories “asymptomatic” and “improvement” were combined to represent better therapeutic success.

Statistical processing was performed using Minitab^®^ Release (software version 14, Minitab, LLC, State College, PA, USA). Descriptive statistics were employed for categorical variables (frequencies) and for quantitative variables (mean, median, and standard deviation). The association between independent variables and clinical outcomes was tested using the Chi-square (χ^2^) test and Analysis of Variance (ANOVA), adopting a significance level of *p* < 0.05.

## 3. Results

According to Table 1, 125 patients were treated: 52% between 2003 and 2014 and 48% between 2015 and 2019, with an average age of 41 years. Women accounted for 76%. After evaluating the main complaint, the most frequently reported possible nerve injuries were the inferior alveolar nerve (IAN) in 76% of patients and the lingual nerve (TN) in 24%. Approximately 7% of individuals had simultaneous paresthesia in both nerves (IAN + TN).

Neurosensory disturbance was the most prevalent after exodontia (60.8%) and orthognathic surgery (13.6%) procedures. This symptomatology was also reported in 12.8% of individuals who suffered some facial trauma. Comorbidity was reported in 66.4% of patients. Regarding the use of medications to assist in the therapy of paresthesia, 22.4% of individuals used them at some point since diagnosis. Orofacial paresthesia was more frequently reported on the left side of patients (46.4%). There were also reports of bilateral numbness in 17.6% of cases—approximately 70% of the treatment was completed.

Regarding the laser light therapy protocol used in the studied population, the average doses were: Dose per session (DS) 88 J/cm^2^ and at the end of the cycle, a therapeutic or cyclic dose (DC) of 1095 J/cm^2^. Around 35 sessions were performed, corresponding to approximately 3 laser therapy cycles performed per patient (Table 1).

**Figure 1:**
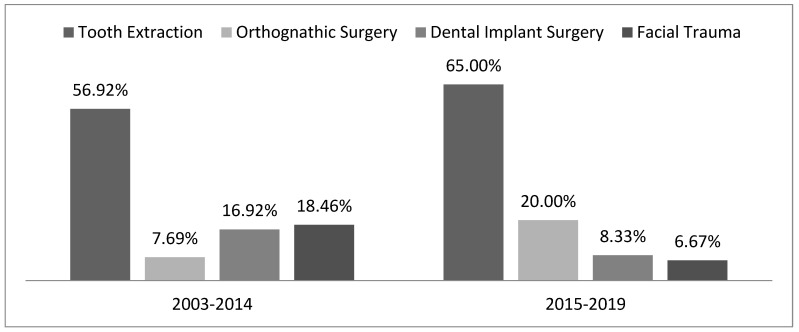
Causes of orofacial neurosensory alterations in patients treated at the Laser Clinic of the Biophotonics Center of FOUFBA, Salvador-BA-Brazil, in the periods from 2003 to 2014 and from 2015 to 2019. (*p* = 0.02—Chi-square).

Figure 1 shows that tooth extraction was the most prevalent cause among treated patients in both periods. In the first period (2003 to 2014), paresthesia was also present in individuals who suffered facial trauma. In the second period (2015 to 2019), also in those who underwent orthognathic surgery (20%), these data were statistically significant (*p* = 0.002).

In the final analysis, approximately 67% of the 125 patients reported improvement in outcomes, including a minimum 4-point change on the inverted Visual Analog Scale (VAS). Orofacial neurosensory alterations persisted in approximately 17% of them, resulting in unsatisfactory outcomes and abandonment in 16%.

When analyzing the association between the studied variables and the better final treatment outcome (Table 2), only the number of completed cycles (78%) was associated with a favorable one, with this relationship being statistically significant (*p* < 0.001).

Both men and women obtained satisfactory results in their groups above 60%, as did the age groups, although without a statistically significant difference (*p* > 0.05, Table 2). There was no significance when it was associated with the use of other medicines.

Regarding the injured nerves, improvement results were also high, at 65.26% for IAN and 76.19% for TN. There were no statistically significant differences in outcomes for the comorbidity variables or the medication-use variable in the treatment of orofacial neurosensory alterations. However, in all groups, these variables showed favorable outcomes (*p* > 0.05; Table 2), although these patterns should be interpreted cautiously within the study design.

**Figure 2:**
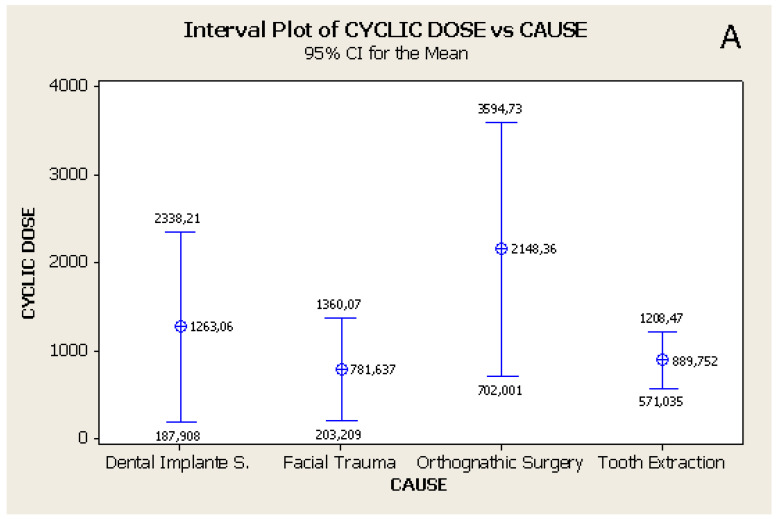
Interval Plot and 95% CI for the mean of cyclic dose X cause.

There was a statistically significant difference (*p* = 0.04) in relation to cyclic doses and possible causes (Figure 2). In individuals who had undergone orthognathic surgery, the average cyclic doses were higher (2148 J/cm^2^) than those who suffered facial trauma (782 J/cm^2^) and exodontia (889.75 J/cm^2^), within the observed treatment protocols. Figure 2: Mean and confidence interval of cyclic doses (J/cm^2^) related to the variable of causes of orofacial neurosensory alterations A) (*p* = 0.04, ANOVA).

## 4. Discussion

The 67.20% clinical improvement was reported among cases that followed a protocol of three weekly sessions at 48 h intervals, with an average cyclic dose (CD) of 1196.7 J/cm. However, this finding should be interpreted with caution. Variations in cyclic dose across etiological groups should be considered in the context of baseline clinical differences, such as severity and type of nerve injury, which were measured solely on patient-reported morbidity, which may have influenced both the therapeutic approach and the distribution of reported outcomes. These factors limit the interpretation of the observed patterns and should be taken into account when analyzing the results.

The adopted therapeutic interval is based on Arndt-Schultz’s Law, as adapted by Arjmand (2021) [14], which advocates a therapeutic window for photostimulation to avoid bioinhibition from overdose. Although there is no consensus on the optimal periodicity, the present findings align with reports of alternating application regimens associated with neuroregenerative responses [10,15,16].

Regarding the physical parameters used in this study, red laser photobiomodulation (typically within the 630–700 nm wavelength range) has been described in the context of orofacial neurosensory alterations, particularly because it interacts more with superficial tissues than infrared wavelengths. This optical characteristic enables energy absorption in the epithelial and subepithelial layers, which may be relevant in cases involving terminal nerve branches or more localized sensory disturbances [17,18,19,20,21,22,23]. From a biological perspective, red light has been associated with cellular photoreception mechanisms involving mitochondrial chromophores, thereby modulating cellular metabolism, ATP production, and local microcirculation. These processes have been described in experimental and clinical literature as potentially related to neural tissue response, including changes in inflammatory mediators and cellular signaling pathways. In this context, red laser has been reported in protocols aimed at sensory modulation and tissue repair [3,13,15,16,24]. These observations contribute to the characterization of therapeutic profiles rather than establishing comparative superiority between wavelengths.

From an etiological perspective, tooth extraction was the predominant causal factor (60.80%), with the inferior alveolar nerve being the most affected structure (76%). This distribution highlights the clinical relevance of postoperative monitoring, particularly in procedures with a higher likelihood of nerve involvement, emphasizing the need for systematic follow-up and standardized assessment of neurosensory function over time [15,21,23,24]. An increase in cases after orthognathic surgery (20%) was also noted between 2015 and 2019, possibly associated with the complexity and duration of these procedures [23,24,25]. This trend may also reflect an expansion in the indication and availability of orthognathic interventions during this period, as well as improvements in referral systems to specialized centers, which can influence the observed distribution of cases. However, the retrospective nature of the data does not allow for differentiation between a true increase in incidence and changes in case capture or reporting patterns.

Cases with unfavorable clinical evolution were descriptively associated with more severe neural injuries, such as neurotmesis, which are typically linked to persistent facial neurosensory disorders [15,22]. Nevertheless, the absence of standardized diagnostic criteria and objective neurosensory testing may limit the precision of this classification, as severity was inferred from clinical records and patient-reported outcomes. In addition, the timing between injury and initiation of treatment—an important factor described in the literature—could not be consistently controlled and may have influenced the observed patterns.

Conversely, the lower frequency of neurosensory alterations following implant procedures (8.33%) may reflect advances in minimally invasive techniques in contemporary implant dentistry, including improved imaging, surgical planning, and operator experience. However, this finding should be interpreted cautiously, as less severe or transient cases may be underreported or not referred to specialized services, potentially leading to an underestimation of their prevalence in this sample [26].

Regarding biological variables, although previous studies suggest that factors such as sex and age may influence neural recovery [27,28], no statistically significant differences were observed between genders or age groups in this sample. The proportion of improvement (73%) among older individuals suggests that increasing age did not preclude a favorable clinical course; however, these findings should be interpreted cautiously and within the descriptive scope of the study. Additionally, potential confounding factors, such as baseline severity, comorbidities, and treatment adherence variability, were not controlled for and may have influenced the observed distribution of outcomes.

The cyclic dose and use of the infrared spectrum described here differ from protocols employing combined wavelengths and lower doses in other neuropathic conditions, such as diabetic neuropathy [29]. This distinction suggests variability in therapeutic approaches across clinical contexts, but it does not allow causal inference about superiority. Additionally, the absence of a statistical association between the use of B-complex vitamins or anti-inflammatory drugs and clinical outcomes [30] should be interpreted as a descriptive finding rather than evidence of a lack of efficacy.

Other factors that could not be measured in this study but are described in the literature include, at the molecular level, photobiomodulation’s association with increased expression of growth factors and mechanisms involved in neurotissue repair [19,30]. In the present study, although detailed information on medication use was limited, no association was identified between pharmacological therapy and clinical evolution. Notably, clinical improvement was observed in approximately 69% of patients who did not receive adjunctive drug therapy, reinforcing the descriptive observation that photobiomodulation is a relevant component in the management of facial neurosensory disorders in this sample.

These findings should be interpreted with caution, given the study design. The retrospective, observational nature of the study, the lack of temporal sequencing, potential confounding factors, and the absence of a control group (due to the use of secondary data) limit the ability to establish temporal or causal relationships between the intervention and the reported outcomes. In addition, the assessment of neurosensory changes was based on patient-reported morbidity, and other factors that could not be measured in this study but are described in the literature may have influenced the observed patterns. Differences in cyclic dose across etiological groups should also be considered alongside baseline clinical characteristics, including variation in case severity.

Overall, the results are limited to descriptive and exploratory interpretations of clinical practice in this setting. Further studies, particularly those with longitudinal designs and appropriate comparison groups, are required to characterize these observations better and support the development of standardized clinical protocols for the management of orofacial neurosensory alterations.

On the other hand, the presentation of clinical cases from this series allowed for a more detailed characterization of the epidemiological context of a capital city in Northeast Brazil. It contributed to improvements in service organization to better meet the demands of its healthcare network. Validation of the photobiomodulation protocol as an adjuvant intervention may represent an essential strategy for reducing healthcare costs, relieving patients of the burden of pharmacological expenses, and optimizing healthcare system resources.

## 5. Conclusions

The findings of this study describe the use of laser photobiomodulation in patients with orofacial neurosensory alterations, with approximately 67% of individuals reporting symptom changes, including partial or complete resolution, following treatment with infrared light. Variations in cyclic dose were observed according to the reported etiology, with individuals undergoing orthognathic surgery presenting higher accumulated doses compared to those with trauma-related conditions.

Furthermore, the use of secondary data and the lack of standardized longitudinal follow-up may restrict the consistency and generalizability of the findings. Overall, the results are limited to descriptive and exploratory interpretations of clinical practice in this setting. Further studies, particularly those with longitudinal designs and appropriate comparison groups, are required to characterize these observations better and support the development of standardized clinical protocols for the management of orofacial neurosensory alterations.

## Figures and Tables

**Table 1:** Characterization of patients with orofacial neurosensory alterations treated at the Laser Clinic of the Biophotonics Center of FOUFBA, Salvador-BA, 2003 to 2019. N = 125.

Variables	N	%
Years		
2003 to 2014	65	52.00
2015 to 2019	60	48.00
Gender		
Female	95	76.00
Male	30	24.00
Injured nerve		
Inferior alveolar (IAN)	95	76.00
Lingual (TN)	21	16.80
Both (IAN + TN)	9	7.20
Etiology		
Tooth Extraction	76	60.80
Orthognathic Surgery	17	13.60
Dental Implant Surgery	16	12.80
Facial Trauma	16	12.80
Affected Side		
Right	45	36.00
Left	58	46.40
Bilateral	22	17.60
Comorbidities		
Yes	83	66.40
No	42	33.60
Medications to control paresthesia		
Yes	28	77.60
No	97	22.40
Cycles Completed		
Complete	88	70.40
Incomplete/abandoned	37	29.60
	Mean	Standard Deviation
Age	41.31	16.21
Session dose (SD) (J/cm^2^)	87.7	128.9
Cyclic Dose (CD) (J/cm^2^)	1095	1740
Total sessions	34.68	34.40
Cycles	2.76	2.85

**Table 2:** Variables associated with the end of treatment in patients with orofacial neurosensory alterations at the Laser Clinic, Biophotonics Center of FOUBA, 2003–2019. N = 125.

	Improvement at the End of Treatment		No Improvement at the End of Treatment		Abandonment		Total	*p* Value
Variables	N	%	N	%	N	%	N	
Gender								
Female	60	63.16	19	20.00	16	16.84	95	0.12
Male	24	80.00	2	6.67	4	13.33	30	
Age								
19 to 37 years of age	41	64.06	12	18.75	11	17.19	64	0.52
38 to 83 years old	43	70.49	9	14.75	9	14.75	61	
Injured nerve								
Inferior alveolar (IAN)	62	65.26	16	16.84	17	17.89	95	0.71
Lingual (TN)	16	76.19	3	14.29	2	9.52	21	
Both (IAN + TN)	6	66.67	2	22.22	1	11.11	9	
Causes								
Tooth Extraction	47	61.84	17	22.37	12	15.79	76	0.17
Orthognathic Surgery	14	82.35	2	11.76	1	5.88	17	
Dental Implant Surgery	11	68.75	2	12.50	3	18.75	16	
Facial Trauma	12	75.00	-	-	4	25.00	16	
Comorbities								
No	27	64.29	6	14.29	9	21.43	42	0.49
Yes	57	68.67	15	18.07	11	13.25	83	
Medications to control paresthesia								
No	67	69.07	14	14.43	16	16.49	97	0.37
Yes	17	60.71	7	25.00	4	14.29	28	
Cycles Completed								
Complete	69	78.47	19	21.59	-	-	88	<0.001
Incomplete/abandoned	15	40.54	2	5.41	20	54.05	37	

## Data Availability

The original data presented in the study are openly available in the repository of the postgraduate program in dentistry and health in https://docs.google.com/spreadsheets/d/1PVTb2lxKxTPy-mx7dYUXfjQHPTLedSnH/edit?usp=sharing&ouid=106213072280853158192&rtpof=true&sd=true (accessed on 20 February 2026).
